# Ginger (*Zingiber officinale *roscoe) extract could upregulate the renal expression of *NRF2* and *TNFα* and prevents ethanol-induced toxicity in rat kidney

**Published:** 2021

**Authors:** Rozita Fathi, Abolfazl Akbari, Khadijeh Nasiri, Marjan Chardahcherik

**Affiliations:** 1 *Department of Exercise Physiology, Faculty of Sport Science, University of Mazandaran, Babolsar, Iran*; 2 *Athletic Performance and Health Research Center, University of Mazandaran, Babolsar, Iran*; 3 *Department of Physiology, School of Veterinary Medicine, Shiraz University, Shiraz, Iran*; 4 *Department of Biochemistry, School of Veterinary Medicine, Shiraz University, Shiraz, Iran*

**Keywords:** Kidney, Ethanol, Ginger, Oxidative stress, NRF2, TNF-α

## Abstract

**Objective:**

Ginger has protective effects on the kidney, however the molecular mechanism of this effect has not yet been fully elucidated. Therefore, this work studied molecular mechanisms of ginger effects on ethanol-induced kidney injury.

**Materials and Methods:**

Twenty-four male Sprague-Dawley rats were randomly divided into four groups: control, ginger (1 g/kg/day ginger extract by oral gavage), ethanol (4 g/kg/day ethanol by oral gavage) and ginger-ethanol group and treated daily for 28 days. Kidney function, expression of nuclear factor erythroid 2-related factor 2 (NRF2) and tumor necrosis factor (TNF)-α genes and oxidative stress parameters in kidney tissue, were evaluated. Total phenolic content (TPC) and 2, 2-diphenyl-1-picrylhydrazyl (DPPH) scavenging activity of ginger extract were also evaluated.

**Results:**

Hydroethanolic extract of ginger showed a good level of DPPH scavenging activity and TPC. In the ethanol group, serum level of urea, creatinine and uric acid and the expression of *NRF2* and *TNF-α* significantly increased compared to control group, while co-treatment with ginger in ginger+ethanol group significantly ameliorated them compared to the ethanol group. Ethanol exposure significantly reduced the activity of superoxide dismutase  (SOD), glutathione peroxidase (GPx) and catalase (CAT) compared to the control values ,while the level of malondialdehyde (MDA) significantly increased. Ginger significantly ameliorated the level of MDA and activity of SOD, GPx and CAT in the ginger-ethanol group compared to the ethanol group.

**Conclusion:**

The results showed that ginger's protective effects against ethanol renotoxicity were mediated via enhancing the *NRF2* and *TNF-α* expression.

## Introduction

Nowadays people's lifestyle is closely linked with exposure to radio waves, chemicals such as toxins and insecticides, and or smoking and alcohol (Akbari et al., 2016 ▶; Akbari et al., 2017 ▶). Alcohol is a common and popular drink and its excessive intake is known as a risk factor of chronic diseases such as liver disease, cancer, diabetes mellitus, reproductive disorders and renal failure (Shield et al., 2013 ▶). Excessive intake of alcohol after smoking and high blood pressure can lead to death in middle and high-income countries. Ethanol is mainly metabolized in the liver and to some extent in kidneys by several enzymes such as cytochrome P450 2E1 (CYP2E1) monooxygenase and  nicotinamide adenine dinucleotide phosphate (NADPH) oxidase (NOX_2_). This metabolism causes the production of acetaldehyde, superoxide (O_2_^•−^) and other reactive oxygen species (ROS). Over production of ROS can cause oxidative stress and damage the cells (Altamirano et al., 2012 ▶). Kidney is a crucial bean-shaped organ involved in formation of urine, hormone secretion, blood pressure regulation, acid-base balance, regulation of osmolality and metabolism. Kidneys are responsible for blood clearance and are constantly exposed to toxic chemicals such as drugs and their metabolites; in addition central parts of the kidneys receive limited blood suply, therefore, they are prone to oxidative damage (George et al., 2017 ▶). Nephrotoxicity or kidney injury is usually detected through evaluating the plasma level of blood urea nitrogen (BUN), creatinine, urea and acid uric (Kim and Moon, 2012 ▶). Evaluation of these parameters is clinically useful to check patients with kidney diseases. Oxidative stress, imbalance between ROS production and the ability of neutralization by antioxidants, along with inflammation, are the most important players in the pathogenesis of chronic renal failure associated with long-term alcohol consumption. Evidence indicated that ROS overproduction, lipid peroxidation, and depletion of antioxidant systems in epithelial tubular cells, are the main pathomechanisms associated with nephrotoxicity induced by ethanol (Latchoumycandane et al., 2014 ▶). NRF2/Keap-1/HO-1 pathway which regulates the cellular redox and phase II detoxification responses, has attracted many researchers' attention in recent years. The NRF2/Keap-1/HO-1 pathway is inactive under physiological conditions but it is activated by ROS overproduction (Chen and Maltagliati, 2018 ▶; Fathi et al., 2020 ▶). Therefore, strengthening the endogenous antioxidant through the use of herbal supplements containing phenolic and flavonoid compounds such as ginger, can prevent the development of many diseases (Nimrouzi et al., 2020a ▶; Jelodar et al., 2020 ▶; Ostovar et al., 2020 ▶).

Ginger (*Zingiber officinale* Roscoe) belongs to the Zingiberaceae family. Rhizome or ginger root is widely used as a spice and a folk medicine worldwide (Ross, 2005 ▶). Ginger contains phytochemical compounds such as flavonoids, phenols and proteins which are used to prevent and treat many disorders including cardiovascular (Nicoll and Henein, 2009 ▶), eye (Akbari et al., 2019a ▶), renal (Rafieian-kopaei, 2013 ▶), reproductive (Akbari et al., 2017 ▶) and hepatic (Akbari et al., 2019b ▶) disorders. Zingerone, gingerdiol, zingiberene, gingerols and shogoals are the main compounds of ginger with antioxidant (Ghasemzadeh et al., 2010 ▶), anti-inflammatory (Lantz et al., 2007 ▶), anti-diabetic and hypo-lipidemic properties (Al-Amin et al., 2006 ▶). Shanmugam et al. (2010) ▶ showed that ginger in a dose-dependent manner (100 and 200 mg/kg) could reverse ethanol-induced oxidative damage in rat kidney (Shanmugam et al., 2010 ▶). Although the therapeutic potential of ginger in these studies (Hamed et al., 2012 ▶, Shanmugam et al., 2010 ▶) was well documented, the molecular mechanisms of such effects have not yet been elucidated. Therefore, the purpose of this study was to investigate the protective effects of ginger extract on ethanol-induced nephrotoxicity and oxidative stress in rats.

## Materials and Methods

**Materials**

Ethanol 96% was purchased from Razi Chemical Company (Tehran, Iran) and other chemicals, reagents and standard solutions used in the study were purchased from Merck (Darmstadt, Germany) and Sigma-Aldrich (St. Louis, MO, USA).

**Preparation of ginger (** ***Zingiber officinale*** **Roscoe) extract**

Rhizome of ginger was purchased from a herbal shop (*Attari*) in Shiraz, Iran. Then, it was verified by a botanist in Shiraz University of Medical Sciences and assigned with the voucher number: PM-948. Afterwards, 250 g of dried ginger powder was mixed with 500 ml of 70% ethanol and water in an Erlenmeyer. It was then filtered using a filter paper after 48 hr and evaporated at 40°C. The residual was the extract of ginger. 

**Evaluation of total phenolic content (TPC) and total antioxidant capacity**

The evaluation of the extract total phenol content was done using modified Folin-Ciocalteu spectrophotometric method as described by Waterhouse (2002) ▶ (Waterhouse, 2002 ▶). 

Total antioxidant capacity of ginger extract was evaluated by Diphenylpicyl hydrazine (DPPH) method as previously described (Leong and Shui, 2002 ▶).

**Animals**

All stages of this research were conducted in accordance with the "Guidelines for the Care and Use of Research Animals" approved by Shiraz University. Twenty-four adult male Sprague-Dawley rats (220±15 g) were housed in an animal room under controlled conditions: lighting (12 hr light: 12 hr darkness) and temperature (20±2ºC); animals had free access to pelleted food and tap water.

**Experimental design**

The protective effects of ginger extract against toxicity induced by ethanol in rat kidney were studied by dividing animals into four groups, each group included six rats. 

Group I: Vehicle or control group which received normal saline (1 ml/day)(Lapin, 1995 ▶, Receno et al., 2018 ▶)

Group II: Ethanol group which received ethanol (4 g/kg of Body Weight (B.W)/day) for 28 consecutive days (Alirezaei et al., 2012 ▶)

Group III: Ginger group was assigned to receive ginger rhizome extract (1 g/kg of B.W/day) (Al-Qudah et al., 2016 ▶)

Group IV: Ginger-ethanol group which received ethanol (4 g/kg of B.W/day) after administration of ginger (1 g/kg of B.W/day) for 28 consecutive days.

The vehicle and the extract of ginger was administered by gavage daily for 28 days.

**Sampling and assessment of kidney function and oxidative status**

Animals were killed by anesthetizing with ether at the end of the study period after a fasting night. Blood samples were collected through heart puncture. After blood clotting, the sera were used to evaluate creatinine and uric acid and urea. Creatinine and uric acid were determined by Jaffe reaction and enzymatic method (uricase), respectively. Urea was measured by diacetyl monoxime method. The right kidney was immediately dissected and rinsed in ice saline. It was then manually homogenized using phosphate buffered saline (0.1 M, pH 7.4), and centrifuged at 3000 g for 10 min. The upper clear supernatants were used for evaluating biochemical and molecular parameters. Total antioxidant capacity of the kidney tissue was evaluated by Ferric Reducing Ability of Plasma (FRAP) method as previously described by (Benzie and Strain, 1999 ▶). The activities of SOD and GPx were measured by detection kit (Ransod and Ransel kits, respectively; RANDOX Company, UK). The level of MDA was evaluated by a modified method as described previously (Alirezaei et al., 2012 ▶). Catalase activity and total protein were determined according to methods described by Aebi (Aebi, 1984 ▶) and Lowry et al. (1951) ▶(Lowry et al., 1951 ▶), respectively. 

**RNA extraction and cDNA synthesis**

The characteristics of primer sequences are presented in Table 1. RNA samples were isolated from rat’s kiddney tissue using the extraction kit (Qiagen RNeasy Mini Kit, Germany) according to manufacturer's instruction. Quantification and qualification of total RNA concentration was estimated using Nanodrop ND-1000 spectrophotometer (Thermo Scientific). Reverse Transcriptase (M-MLV RT) (Yekta Tajhiz Azma*,* Iran) and Oligo-dT primer for the synthesis of cDNA were used. From each sample, 2 μg of total RNA was used to synthesize cDNA. In this study, *TNFα* and *GAPDH* primers were designed using Primer Premeir 5 software and *NRF2* primers were used from the study by Liang et al. (2017).

Determination of relative quantity in Real Time PCR was done by measuring the increase of fluorescence light, as the result of attaching SYBR Green color to DNA. During this step, the Polymerase chain Reaction was performed on cDNA samples to amplify *NRF2* and *TNFα* genes and *GAPDH* as the reference gene, using RealQ Plus 2x Master Mix Green (Ampliqon, Denmark) in Rotor Gene 6000 (Corbett Research, Australia). Real Time PCR reactions were performed in a final volume of 20 μl and each reaction was duplicated. The reaction mixture contained 3 μl of cDNA (50 ng/μl), 8 μl of RealQ Plus 2x Master Mix Green, 0.4 μl of each of primers (10 pmol) and 8.2 μl of ribonuclease-free water. The temperature program was as follows: enzyme activation 95°C for 13 min, followed by 40 cycles of denaturation at 95°C for 30 sec, primer annealing at 58°C (GAPDH) and 60°C (NRF2 and TNFα) for 35 sec and extension at 72°C for 30 sec, melting curve at 60°C for 5 sec, and the final step at 95°C as continuous; 2^-^^∆∆^^CT^ method was used to calculate the fold change in genes expression (Rao et al., 2013 ▶).

**Histopathological examination**

Kidney was removed and fixed in a 10% formalin solution for at least three weeks and was then embedded in paraffin. After that, sections were cut at 5 μm and stained with hematoxylin and eosin. These sections were then examined under a light microscope (×40, BX-51, Olympus Corporation, Tokyo, Japan) for evaluating the degenerative changes in tubules and congestion in glomerular capillaries and medullary vessels in rat kidney**.**

**Statistical analysis**

The results were analyzed using one-way analysis of variance (ANOVA) followed by *post hoc* multiple comparisons Tukey test to compare the results of different treatment groups. All data were recorded using Statistical Package for Social Sciences (SPSS 17.0). The descriptive results are expressed as means±standard error of mean (Mean±SEM). The statistical significance was set at p<0.05.

**Table 1:** The characterizations of primer sequences used in this study

**Tissue**	**Gene**	**Primer sequences**	**Product length (bp)**
Kiddney	*NRF2*	Forward 5´-GCTGCCATTAGTCAGTCGCTCTC-3´ Reverse 5´-ACCGTGCCTTCAGTGTGCTTC-3´	104
	*TNFα*	Forward 5'- CTT CAG GGA TAT GTG ATG GAC TC-3' Reverse 5'- GGA GAC CTC TGG GGA GAT GT -3'	186
	*GAPDH*	Forward 5´-GGCAAGTTCAACGGCACAG-3´ Reverse 5´-GACGCCAGTAGACTCCACGAC-3´	144

## Results

**Figure 1:**
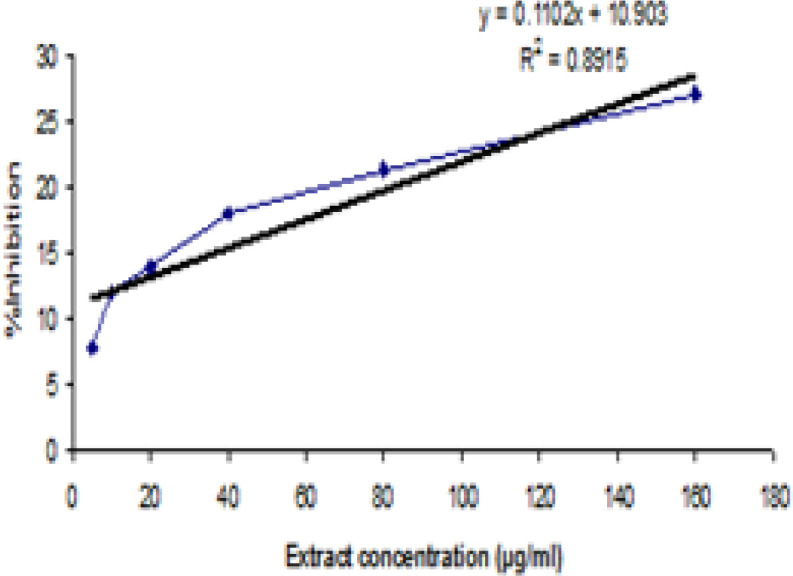
DPPH radical scavenging activity of extract of ginger

The results of TAC of extract of ginger are presented in Figure 1. The results showed that the ginger extract exhibited a good DPPH scavenging activity (IC50 = 354.782 mµ/mL). In addition, the TPC in ginger extract was calculated from equation of calibration curve and was expressed as milligrams of gallic acid equivalents per gram of dry extract (mg of GAE/g of dE). It was 89.84mg GAE/g dE.

The serum level of urea, creatinine and uric acid was measured to evaluate kidney function (Table 2). In the ethanol group, the serum level of urea, cratinine and uric acid significantly increased compared to other groups (p<0.01, Table 2); while, the level of these parameters in the ginger-ethanol group was significantly lower compared to the ethanol group (p<0.05, Table 2).

**Figure 2:**
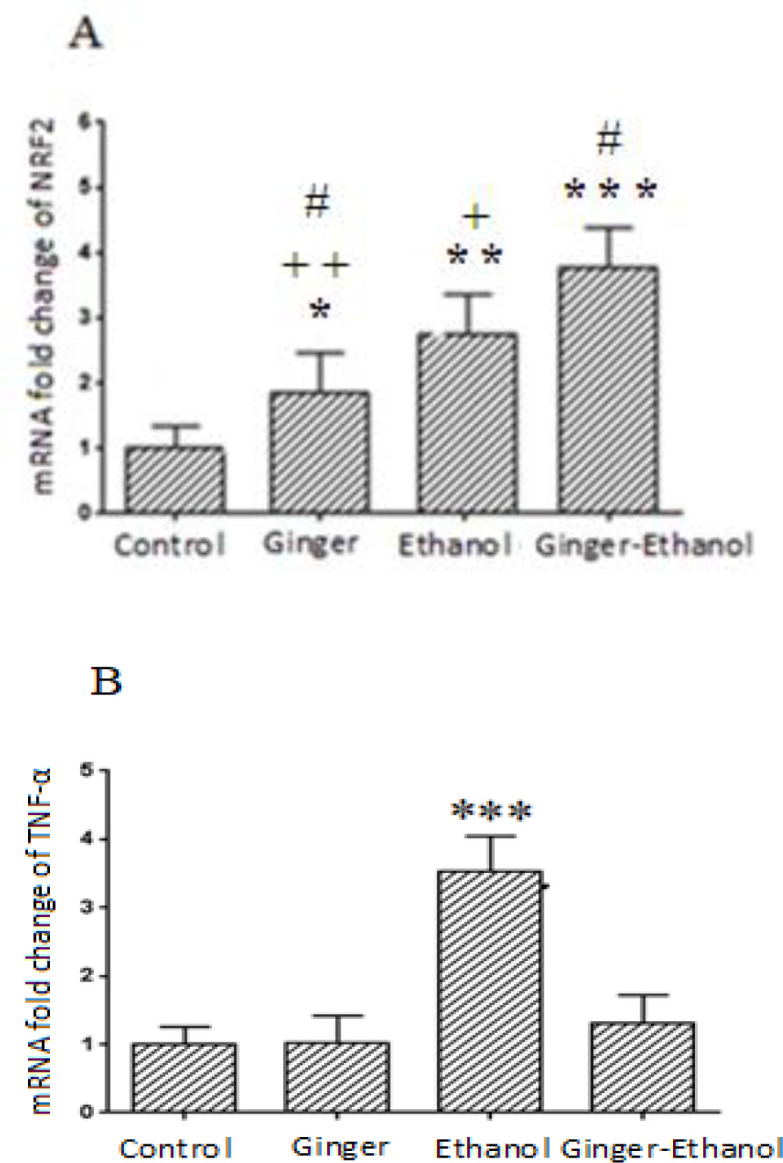
Comparison of the expression of *NRF2* (A) and *TNFα* (B) among control and treated groups (n=6). Data are expressed as mean±SEM

The renal expression of *NRF2* and *TNFα* genes is presented in Figure 2. Our results showed that renal expression of *TNFα* gene was significantly higher in the ethanol group compared to other groups, while co-treatment with ginger significantly decreased expression of this gene in the ginger-ethanol group compared to the ethanol group (p<0.001, Figure 2B). The results also showed that there is no significant difference between ginger and control group regarding the experssion of this gene (Figure 2B).

Our results also indicated that renal expression of *NRF2* significantly increased in ginger, ethanol and ginger-ethanol groups compared to the control (p<0.05 to p<0.001, Figure 2A). The renal expression of this gene significantly increased in the ethanol group compared to the control and ginger groups. Moreover, in the ginger group, the expression of *NRF2* was higher than the control but lower than the ethanol and ginger-ethanol groups. The results also showed that the *NRF2* gene expression has the highest level in the ginger-ethanol group compared to other groups (p<0.05, Figure 2A). 

A. *p<0.05, **p<0.01 and ***p<0.001 significant difference compared to the control group. +p<0.05, ++p<0.01: significant difference compared to the Ginger-Ethanol group. #p<0.05: significant difference compared to the ethanol group.

B. ***p<0.001 vs.other groups.

**Figure 3:**
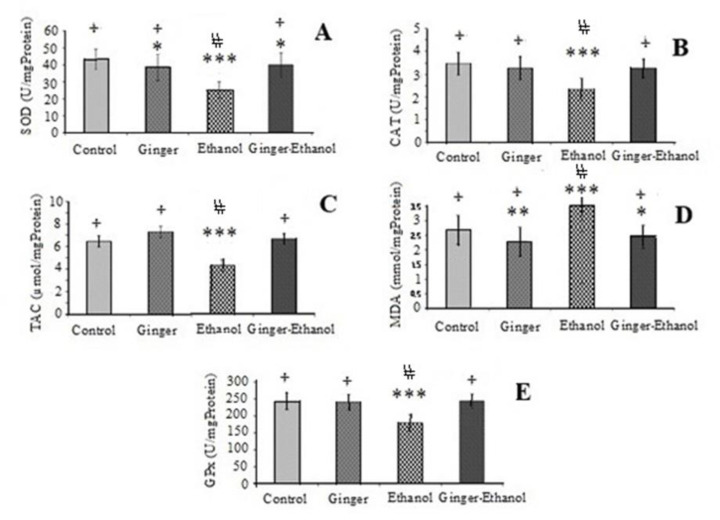
Comparison of SOD (superoxide dismutase. A), CAT (catalase, B), TAC (total antioxidant capacity, C), MDA (Malondialdehyde, D) and GPx (glutathione peroxidase, E) among different groups (n=6). Data are expressed as mean±SEM

The protective effect of ginger on kidney toxicity induced by ethanol was evaluated by monitoring the renal level of SOD, GPx and CAT activity and the level of TAC and MDA. The mean value (±SEM) of these parameters is presented in Figure 3. Based on our results, the activity of SOD, GPx and CAT and the level of TAC in the ethanol group significantly decreased compared to control group (p<0.001, Figure 3). While, the administration of ginger could increase these parameters in the ginger-ethanol group compared to the ethanol group (p<0.05, Figure 3). Additionally, the increased level of MDA by alcohol, was reduced through co-treatment with ginger (p<0.05, Figure 3).

**Figure 4:**
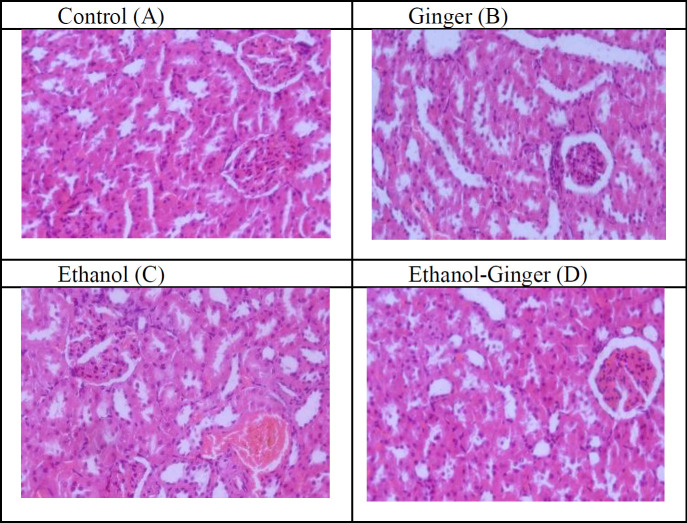
Histological finding of the rat kidney sections in the studied groups (×40). Light microscopic examination of kidney sections from control (A) and ginger (B) groups showed normal arrangement of glomerulus and no congestion in glomerular capillaries and medullary vessels. Evaluation of kidney sections from the ethanol group (C) showed that ethanol induced injury in the kidney which is characterized by abnormal glomerulus and congestion in glomerular capillaries, while co-treatment with ginger in animals treated with ethanol, improved these injuries (D)

The histological finding of the rat kidney sections in the studied groups, is presented in Figure 4. Light microscopic examination of kidney sections from control and ginger groups showed that there were no congestion in glomerular capillaries and medullary vessels (Figure 4A and 4B). The evaluation of kidney sections from the ethanol group showed that ethanol induced congestion in glomerular capillaries and medullary vessels (Figure 4C), while co-treatment with ginger in animals receiving ethanol, significantly improved these abnormalities in the cortex and medulla of kidney (Figure 4D). 

**Table 2:** The mean value (±SEM) for urea, cratinine and uric acid in studied groups

**Group**	**Control**	**Ginger**	**Ethanol**	**Ethanol-Ginger**
Urea (mg/dl)	14.15±1.36	15.78±1.45	28.8±1.67*	16.5±1.14
Creatinine (mg/dl)	0.46±0.12	0.48±0.07	1.73±0.18^#^	1.06±0.15*
Uric acid (mg/dl)	6.15±0.47	6.94±0.39	11.5±0.44*	6.96±0.49

* p<0.05 vs. other groups, # p<0.01 vs. control and ginger groups.

*p<0.05, **p<0.01 and ***p<0.001 significant difference compared to the control group. +p<0.001 significant difference compared to the ethanol group. #p<0.001 significant difference compared to the ginger-ethanol group.

## Discussion

In the current study, the serum level of urea, creatinine and uric acid as the indices of kidney function, increased by ethanol which is in agreement with previous reports (Altamirano et al., 2012 ▶; Latchoumycandane et al., 2014 ▶; Shirpoor et al., 2016 ▶). The results also showed that the level of these parameters improved by pre-treatment with ginger. Shanmugam et al. (2010) ▶ showed that ethanol (2 g/kg body weight, once daily for 30 days) induces oxidative stress and changes kidney tissue, while treatment with ethanolic extract of ginger (100 and 200 mg/kg body weight, once daily for 30 days) return these parameters to normal levels (Shanmugam et al., 2010 ▶). Moreover, Shirpoor et al. (2016) ▶ showed that ethanol induces oxidative DNA damage, functional and structural changes in kidney of rats, meanwhile ginger alleviated functional and structural alterations in kidney of rats (Shirpoor et al., 2016 ▶). However, Latchoumycandane et al. (2014) ▶ stated that ethanol-induced kidney dysfunction correlates with leukocyte infiltration and activation, and not primarily from metabolism of ethanol by CYP2E1. CYP2E1 metabolism may be necessary to initiate inflammation in the liver (Altamirano et al., 2012 ▶), but kidneys contain significantly less of this monooxygenase than the liver, and the liver was only modestly affected by ethanol in this animal model. Conversely, other researchers discovered that neutrophils were abundant in kidneys after ethanol feeding. Neutrophil type 2 NADPH oxidase has a critical role in ethanol-induced liver damage (Kono et al., 2001 ▶; Kono et al., 2000 ▶), and may contribute to oxidative stress in kidneys as well (Latchoumycandane et al., 2014 ▶). Latchoumycandane et al., (2014) ▶ suggested that ethanol acts as an indirect nephrotoxin to induce Acute Kidney Injury. Chronic ethanol metabolism induces an unappreciated cycle of leukocyte infiltration and activation necessary to induce its nephrotoxic effects. However, we did not evaluate the status and activity of leukocytes in kidneys in our study, nor did they evaluate the activity of renal antioxidant and inflammatory system. In fact, the major contradiction between this study and our study is that we hypothesized that ethanol directly induced kidney damage via oxidative stress and inflammation. In addition, regular ethanol intake increases the blood pressure, which is a risk factor for kidney damage. It should be also noted that a very large volume of blood circulates in the kidneys daily, and the role of these cells (leukocyte and neutrophil) cannot be considered in inducing renal injury. However, strengthening anti-inflammatory and antioxidant systems of the kidneys is essential to improve their function. Accordingly, we studied the expression of *NRF2* and *TNFα* genes along with oxidative status in kidneys. Our results showed that the renal expression of *TNFα* and *NRF2* was increased by ethanol which is in agreement with previous reports (Dong et al., 2008 ▶; Perrien et al., 2003 ▶; Luedemann et al., 2005 ▶; Shirpoor et al., 2016 ▶). The results also showed that the expression of *TNFα* increased by ethanol, while co-treatment with ginger could decrease its expression in rat kidney. TNFα is a cell signaling protein which is involved in systemic inflammation. Moreover, it was reported that ethanol could up-regulate the level of type 1 TNF-receptor (TNF-R1) in different cells and may augment TNF-α-mediated cell injury in different tissues (Rodriguez et al., 2004 ▶). Co-treatment with ginger could decrease the expression of *TNFα* in kidneys. Studies showed that co-treatment and treatment with ginger could reduce the level of TNFα in patients with type 2 diabetes (Mahluji et al., 2013 ▶) and tuberculosis (Kulkarni and Deshpande, 2016 ▶). In addition, Luettig et al. (2016) ▶ indicated that ginger by inhibiting the PI3K/Akt and NF-κB signaling prevents inflammation (Luettig et al., 2016 ▶). Isa et al. (2008) ▶ stated that 6-shogaol and 6-gingerol as ginger ingredients could inhibit TNFα signaling in 3T3-L1 adipocytes (Isa et al., 2008 ▶). Grzanna et al. (2004) ▶ showed that ginger has an effect on several genes encoding cytokines, chemokines and the inducible enzyme cyclo-oxygenase-2 (COX-2) (Grzanna et al., 2004 ▶). To sum up, in addition to down-regulating TNFα expression, ginger is able to inhibit synthesis of pro-inflammatory cytokines such as IL-1 and IL-8 (Tjendraputra et al., 2001 ▶) and NF-κB signaling pathways (Saedisomeolia et al., 2019 ▶). NF-*κ*B plays a key role in activating subsequent signaling pathways, especially the regulation of pro-inflammatory molecules (Mashhadi et al., 2013 ▶). 

The results of this study showed that the level of SOD, TAC, CAT and GPx decreased and the level of MDA increased by ethanol, while co-treatment with ginger could improve these levels which is in agreement with previous reports (Albano, 2006 ▶; Akbari et al., 2017 ▶; Ilkhanizadeh et al., 2016 ▶; Shanmugam et al., 2010 ▶). 

Many studies demonstrated that ginger extract reduce lipid proxidation and oxidative damage induced by ethanol (Akbari et al., 2017 ▶; Shanmugam et al., 2010 ▶), streptozocin-induced diabetes (Ilkhanizadeh et al., 2016 ▶), CCL_4_ (Hamed et al., 2012 ▶), lead (Reddy et al., 2014 ▶) and iron (Gholampour et al., 2017 ▶) induced renal toxicity in male rats. The mechanisms involved in the induction of oxidative stress by ethanol are well-known (Luo et al., 2018 ▶; Lu and Cederbaum, 2008 ▶). Ethanol is a nephrotoxin that acts through a cycle of leukocyte infiltration and activation and ROS production (Latchoumycandane et al., 2014 ▶). Our results showed that the expression of *NRF2* was up-regulated by ethanol and ginger. It was well reported that changes in ROS production are one of the most important stimuli for expression of this gene. The level of ROS was increased by ethanol, therefore it can up-regulate the expression of NRF2. However, the question is how the expression of this gene increased in healthy animals using ginger. It is likely that ginger constituents such as 6-shogaol and 6-gingerol were able to increase the expression of *NRF2* gene through alterations in cellular signaling pathways (Bak et al., 2012 ▶; Schadich et al., 2016 ▶). Interestingly, the expression of this gene was significantly higher in ginger-ethanol group than the ethanol and ginger groups. In fact, this increase in expression is a potent response to the fight against ethanol damage. As previously mentioned, NRF2 is able to inhibit inflammation (Luo et al., 2018 ▶). Loboda et al. (2016) ▶ stated that NRF2, as a cytoprotective factor, not only regulates the expression of genes coding for anti-oxidant, anti-inflammatory and detoxifying proteins, but it is also a powerful modulator of species longevity (Loboda et al., 2016 ▶). Studies showed that NRF2/HO-1 pathway plays a protective role in oxidative and inflammatory responses induced by ethanol (Xu et al., 2018 ▶) and inhibits apoptosis in HEI193 Schwann cells (Jeong et al., 2019 ▶). Moreover, NRF2/Keap-1/HO-1 pathway has an crucial role in preventing the development of chronic diseases by inhibiting oxidative damage and infllamation (Nimrouzi et al., 2020b ▶; Tu et al., 2019 ▶). According to our results, co-treatment with ginger could improve oxidative damage and histological damages induced by ethanol in the kidney which is in agreement with previous reports (Shanmugam et al., 2010 ▶, Hamed et al., 2012 ▶, Gholampour et al., 2017 ▶). Gholampour et al. (2017) ▶ showed that pretreatment with ginger improves the congestion in glomerular capillaries and medullary vessels induced by iron in rat (Gholampour et al., 2017 ▶). Shanmugam et al. (2010) ▶ showed that ethanol induces degenerative changes in tubules, diffused cellular infiltration and congestion of blood vessels in rat kidney, while treatment with ginger (2 g/kg body weight, once daily for 30 days) improved the damage caused by ethanol (Shanmugam et al., 2010 ▶). In fact, the cause of these injuries can simply be considered oxidative damage and inflammation. It is well-known that high levels of ROS react with lipids, proteins and DNA, leading to cells and tissues injury (Akbari et al., 2014 ▶). Lipid peroxidation as a physiological process in membrane, and MDA as a marker for lipid peroxidation is increased by high levels of ROS. Excessive ethanol intake induces structural and functional changes in kidneys and plays a central role in the development of chronic renal failure (Epstein, 1997 ▶). Structural injuries might be associated with the absorption potential of renal tubules after ethanol exposure (Cigremis et al., 2006 ▶). Ginger treatment significantly ameliorates the injurious effects of ethnaol on renal morphology. Previous research showed that ginger had reno-protective effect against CCL_4_ and alcohol (Shanmugam et al., 2010 ▶; Hamed et al., 2012 ▶). Besides, the protective effects of ginger against damage induced by ethanol can be due to antioxidant and anti-inflammatory activity or improvement of trace elements such as zinc (Akbari et al., 2019a ▶; Akbari et al., 2017 ▶). Ginger contains high levels of gingerdiol, zingerone, shogoals, gingerols and zingiberene which inhibit oxidative damage (Semwal et al., 2015 ▶). Our study confirms that ginger precludes the toxic impact of ethanol against kidney damage at both histological and biochemical levels.

The findings of this study indicated that ethanol induced renal oxidative damage and ginger could improve all of these condition. It should be noted that ginger extract due to its role as an antioxidant and its interference with other processes such as homeostasis of essential elements and inflammation, could improve the damages caused by ethanol. 
